# The Expanding Frontier: The Role of Artificial Intelligence in Pediatric Neuroradiology

**DOI:** 10.3390/children12091127

**Published:** 2025-08-27

**Authors:** Alessia Guarnera, Antonio Napolitano, Flavia Liporace, Fabio Marconi, Maria Camilla Rossi-Espagnet, Carlo Gandolfo, Andrea Romano, Alessandro Bozzao, Daniela Longo

**Affiliations:** 1 Functional and Interventional Neuroradiology Unit, Bambino Gesù Children’s Hospital, IRCCS (Istituto di Ricovero e Cura a Carattere Scientifico), 00165 Rome, Italy; fabio.marconi@opbg.net (F.M.); mcamilla.rossi@opbg.net (M.C.R.-E.); carlo.gandolfo@opbg.net (C.G.); daniela.longo@opbg.net (D.L.); 2 Neuroradiology Unit, NESMOS (Neuroscience, Mental Health and Sensory Organs) Department, Sant’Andrea Hospital, La Sapienza University, Via di Grottarossa, 1035-1039, 00189 Rome, Italy; andrea.romano@uniroma1.it (A.R.); alessandro.bozzao@uniroma1.it (A.B.); 3 Medical Physics Department, Bambino Gesù Children’s Hospital, 00165 Rome, Italy; antonio.napolitano@opbg.net (A.N.); flavia.liporace@opbg.net (F.L.)

**Keywords:** artificial intelligence, deep learning, machine learning, pediatric imaging, pediatric neuroradiology, workflow optimization, synthetic imaging, segmentation, MELD, federated learning

## Abstract

Artificial intelligence (AI) is revolutionarily shaping the entire landscape of medicine and particularly the privileged field of radiology, since it produces a significant amount of data, namely, images. Currently, AI implementation in radiology is continuously increasing, from automating image analysis to enhancing workflow management, and specifically, pediatric neuroradiology is emerging as an expanding frontier. Pediatric neuroradiology presents unique opportunities and challenges since neonates’ and small children’s brains are continuously developing, with age-specific changes in terms of anatomy, physiology, and disease presentation. By enhancing diagnostic accuracy, reducing reporting times, and enabling earlier intervention, AI has the potential to significantly impact clinical practice and patients’ quality of life and outcomes. For instance, AI reduces MRI and CT scanner time by employing advanced deep learning (DL) algorithms to accelerate image acquisition through compressed sensing and undersampling, and to enhance image reconstruction by denoising and super-resolving low-quality datasets, thereby producing diagnostic-quality images with significantly fewer data points and in a shorter timeframe. Furthermore, as healthcare systems become increasingly burdened by rising demands and limited radiology workforce capacity, AI offers a practical solution to support clinical decision-making, particularly in institutions where pediatric neuroradiology is limited. For example, the MELD (Multicenter Epilepsy Lesion Detection) algorithm is specifically designed to help radiologists find focal cortical dysplasias (FCDs), which are a common cause of drug-resistant epilepsy. It works by analyzing a patient’s MRI scan and comparing a wide range of features—such as cortical thickness and folding patterns—to a large database of scans from both healthy individuals and epilepsy patients. By identifying subtle deviations from normal brain anatomy, the MELD graph algorithm can highlight potential lesions that are often missed by the human eye, which is a critical step in identifying patients who could benefit from life-changing epilepsy surgery. On the other hand, the integration of AI into pediatric neuroradiology faces technical and ethical challenges, such as data scarcity and ethical and legal restrictions on pediatric data sharing, that complicate the development of robust and generalizable AI models. Moreover, many radiologists remain sceptical of AI’s interpretability and reliability, and there are also important medico-legal questions around responsibility and liability when AI systems are involved in clinical decision-making. Future promising perspectives to overcome these concerns are represented by federated learning and collaborative research and AI development, which require technological innovation and multidisciplinary collaboration between neuroradiologists, data scientists, ethicists, and pediatricians. The paper aims to address: (1) current applications of AI in pediatric neuroradiology; (2) current challenges and ethical considerations related to AI implementation in pediatric neuroradiology; and (3) future opportunities in the clinical and educational pediatric neuroradiology field. AI in pediatric neuroradiology is not meant to replace neuroradiologists, but to amplify human intellect and extend our capacity to diagnose, prognosticate, and treat with unprecedented precision and speed.

## 1. Introduction

Artificial intelligence (AI) and particularly machine learning (ML) and deep learning (DL) are revolutionarily shaping the entire landscape of medical imaging. In this futuristic scenario, radiology represents a privileged medical field since it produces a significant amount of data, namely, images. Currently, AI implementations in radiology are huge and extend from automating image analysis to enhancing workflow management [1], but AI-driven advances are not equally distributed among the subfields. While multiple AI applications in adult imaging have been shown, pediatric subspecialties, particularly pediatric neuroradiology, are now emerging as crucial frontiers where AI can significantly impact clinical practice [2]. A systematic review demonstrated that children represent just under 1% of available data in public medical imaging datasets. This issue is pivotal and shows the need for pediatric-specific big data and AI models [3].

Pediatric neuroradiology presents unique opportunities and challenges since neonates’ and small children’s brains are continuously developing, with age-specific changes in anatomy, physiology, and disease presentation. Therefore, the interpretation of pediatric neuroimaging requires specific knowledge of normal brain development and possible abnormalities. Furthermore, many pediatric neurological conditions are rare, further complicating diagnosis and management [4,5]. Despite these challenges, the potential benefits of AI in pediatric neuroradiology are substantial and early diagnosis is paramount since it may avoid lifelong consequences for neurodevelopmental outcomes.

AI holds tremendous promise in addressing many neuroradiology challenges, such as congenital brain malformations, epilepsy, brain tumours, metabolic and genetic disorders, traumatic brain injury, and perinatal hypoxic-ischaemic injuries [6]. By enhancing diagnostic accuracy, reducing interpretation times, and enabling earlier intervention, AI has the potential to significantly improve patient outcomes. Furthermore, as healthcare systems become increasingly burdened by rising demands and limited radiology workforce capacity, AI offers a scalable solution to support clinical decision-making, particularly in underserved regions where access to specialized pediatric neuroradiologists is limited [7,8].

However, there are several limitations in the integration of AI into pediatric neuroradiology, such as the scarcity of large, high-quality, annotated pediatric imaging datasets and ethical and legal restrictions on pediatric data sharing, that complicate the development of robust and generalizable AI models. Pediatric imaging protocols vary significantly between institutions, and normative data for children are inherently more variable than for adults due to the rapid developmental changes that occur during childhood. As a result, AI tools trained on adult data often fail to generalize to pediatric populations, necessitating the creation of pediatric-specific AI frameworks [1]. Moreover, many radiologists remain sceptical of AI’s interpretability and reliability, especially when used in high-stakes diagnostic scenarios involving young children. There are also important medico-legal questions around responsibility and liability when AI systems are involved in clinical decision-making. Overcoming these concerns will require not only technological innovation but also robust validation studies, regulatory clarity, and multidisciplinary collaboration between neuroradiologists, data scientists, ethicists, and pediatricians [2].

Therefore, AI integration to pediatric neuroradiology has to be human-centred, intelligible, standardized, and supervised to effectively benefit pediatric patients and neuroradiologists and respect the principles of diversity, equity, inclusion and data safety.

The paper aims to address: (1) current applications of AI in pediatric neuroradiology; (2) current challenges and ethical considerations related to AI implementation in pediatric neuroradiology; (3) future opportunities in the clinical and educational pediatric neuroradiology field. 

## 2. Current Applications of AI in Pediatric Neuroradiology

### 2.1. AI-Powered Workflow Management in Pediatric Neuroradiology

Workflow management is a challenging organizational field in healthcare, encompassing various tasks from pediatric patients’ triage to exam scheduling and report prioritizing, with deep reverberations on institutional outcomes, workers’ wellbeing, and patients’ health. In this scenario, AI is increasingly being implemented to automate, improve, and speed up workflow management and enhance operational efficiency [1,7,8,9,10,11]. By automating routine tasks, providing intelligent clinical decision support, and optimizing resource allocation, AI can contribute to improved efficiency, diagnostic accuracy, and ultimately, better patient outcomes [11].

#### 2.1.1. Triage in the Emergency Setting

First Aid is a melting pot of emergencies, urgencies, and anxiety-filled patients who need to be visited, triaged, and receive proper care. In this fast-paced environment, rapid identification and prioritization of critical cases is the everyday challenge. AI may properly support triage workers through algorithms that automatically or semi-automatically analyze patients’ demographic, clinical, laboratory, and anamnestic data to define the severity of disease and patients’ priority in the bigger picture of waiting patients [1,7,9]. Furthermore, AI can analyze a combination of factors, including vital signs, chief complaints, and medical history, to predict the need for critical care or hospitalization in pediatric patients presenting to the emergency department, outperforming conventional triage methods [7,9].

#### 2.1.2. Exam Scheduling

Efficient exam scheduling optimizes resources and minimizes patient waiting times in pediatric neuroradiology departments. AI-powered tools analyze patients’ demographic, imaging, clinical, and laboratory data to produce an efficient worklist. Moreover, AI algorithms showed exceptional accuracy in predicting patients’ flow patterns, the likelihood of patients missing appointments, allowing for proactive interventions to avoid no-shows, and improving overall scheduling efficiency. Particularly, AI-supported notification systems include vocal or text-based notifications 24 h before the appointment as reminders or for appointment confirmation, as well as real-time chat boxes, offering flexibility to reschedule imaging exams due to cancellations or delays, thereby enhancing patients’ and families’ healthcare experience [1].

#### 2.1.3. Imaging Protocol Optimization, Image Enhancement, and Synthetic Imaging

**Figure 1:**
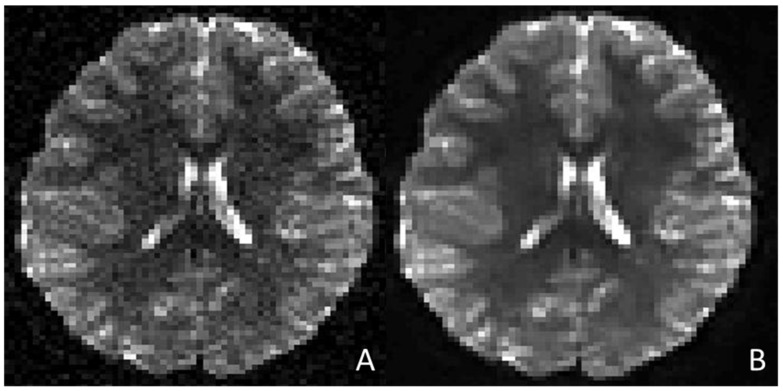
Compares a DTI axial sequence (**A**) to the same DTI sequence after the application of a deep convolutional neural network for denoising purposes [24]. The AI-enhanced DTI (**B**) shows improved quality and decreased noise artefacts (required time 1.1056 s). The network was applied to the data, and the results were visualized using MATLAB (MATLAB ver. 24.1–R2024a [The MathWorks Inc. (2024a). MATLAB version: 24.1 (R2024a), Natick, MA, USA: The MathWorks Inc. https://www.mathworks.com (accessed on 20 July 2025)]). *DTI (Diffusion Tensor Imaging)*.

Optimizing the allocation of Imaging scanners and the selection of the optimal imaging protocol for pediatric patients is critical to ensure high-quality exams, reduce scanner time, and minimize radiation exposure [1,9,12]. Based on patient-specific characteristics, such as age, size, and clinical history, AI algorithms may suggest the most appropriate protocol. CT and X-Ray protocols are specifically tailored to pediatric patients’ demographic, clinical and laboratory data to reduce radiation exposure, and most MRI protocols are built to reduce scanning time and potentially avoid patients’ sedation. In fact, young pediatric patients and neonates or less-cooperative patients cannot stand still during time-consuming exams, such as brain and spine MRIs; therefore, it is crucial to choose the key sequences to avoid or reduce the time of sedation/anesthesia and artefacts [13]. Furthermore, AI-supported guidance on patient positioning, contrast dosing, and image sequencing can improve overall image quality, potentially decreasing the necessity for repeat scans and further limiting radiation exposure [1,9,12]. Moreover, AI-based post-processing algorithms can analyze and increase the quality of ultra-low-dose CT and low-quality MRI exams in order to offer high-quality diagnostic images to neuroradiologists (Figure 1). In pediatric CT, deep learning models and convolutional neural networks (CNNs) have shown substantial improvements in noise reduction and artefact removal, enabling high-quality diagnostic images even by reducing the radiation dose by 36–70% without losing diagnostic information [1,14,15]. AI can also be used to boost contrast in low-iodine-dose CT protocols, which is particularly beneficial in children to minimize the risk of contrast-induced nephropathy [16]. Particularly, the use of contrast agents in pediatric patients raises important concerns, including potential toxicity, especially in cases of repeated exposure, and deposition of contrast agents such as gadolinium in the brain and soft tissues. Moreover, the use of contrast agents may be impossible in cases of severe allergies and impaired renal function. It is crucial to propose AI-driven innovative strategies to avoid or drastically reduce the administration of gadolinium while maintaining the diagnostic value of the images. Specifically, with encoder–decoder DL models, it is possible to synthesize full-contrast MRI images from pre-contrast images by injecting only 10% of the standard gadolinium dose [17]. In MRI, AI methodologies enhance ultra-low-field MRI quality by applying deep learning-based reconstruction schemes to fully or undersampled k-space data, resulting in improved or preserved image quality in equivalent or reduced acquisition times, potentially increasing accessibility to this lower-cost imaging modality [18,19,20].

Synthetic imaging is a novel frontier, characterized by the possibility of creating neuroradiological images from a limited set of acquired data, or even from data acquired by a different imaging modality. The most promising application is synthetic MRI, in which a standardized set of sequences is obtained from a single rapid acquisition, significantly reducing scan times [21]. Moreover, intermodality synthesis allows AI to generate synthetic CT images from MRI data, which can be used for dose calculation in radiotherapy, avoiding additional radiation exposure from a dedicated CT scan [22]. Similarly, AI models can generate synthetic PET images from contrast-enhanced MRI, showing a strong correlation with real PET images for glioma grading and prognostication [23].

#### 2.1.4. Exam Prioritization and Report Generation

Exam prioritization is an AI-powered system that analyses acquired X-ray/CT/MRI images and flags studies characterized by potentially life-threatening findings, such as intracranial hemorrhages, and elevates them to the top of the radiologist’s worklist for immediate review, facilitating timely clinical decision-making and interventions [1,7]. For instance, AI algorithms can detect high-density lesions, suggestive of intracranial hemorrhages on CT scans, with high sensitivity (up to 95%) and specificity (up to 94%), significantly prioritizing the scans and reducing the time to diagnosis. This intelligent exam prioritization ensures that pediatric neuroradiologists focus their attention on the most urgent cases, potentially leading to earlier interventions and improved patient outcomes [7]. To furtherly speed up the healthcare process, AI-based algorithms analyze images and generate preliminary reports including clinically relevant labels and automated impressions of findings, which can then be reviewed and finalized by the radiologist, saving valuable time and reducing cognitive burden [1,7]. Moreover, AI can support the standardization of reports by offering a standard template to neuroradiologists, who are required to fill in the gaps with the required data and suggest a diagnosis. Apart from reducing reporting time, this opportunity is paramount because it paves the way to a global, standardized, readable, and understandable way of reporting imaging exams that avoids misunderstandings and simplifies the following treatment management. The continuous AI-driven worklist re-analysis and re-prioritization dynamically shape the exam and reporting prioritization, which has been demonstrated to significantly reduce report turnaround times for critical conditions [7].

#### 2.1.5. Communication of Urgent and Unexpected Findings

Perfectly scheduling exams, optimizing imaging protocols, exam and report prioritization, and identifying the proper diagnosis are key links of a chain, whose last ring is the diagnosis communication to the referring physician and patients’ or families [25]. Communication in healthcare is key to optimal workflow management. Effective and timely communication of urgent and unexpected findings is paramount in pediatric neuroradiology to ensure appropriate and prompt patient management [26,27,28]. AI plays a vital role in workflow management by ensuring that radiology reports highlight critical findings and are delivered to the relevant referring physicians without delay. AI-driven communication platforms facilitate seamless information sharing and collaboration among the multidisciplinary team involved in the care of pediatric patients. Machine learning approaches can identify and flag radiology reports containing urgent findings that require prompt communication to referring physicians, further enhancing patient safety [1]. Although AI-powered tools retain a significant role in efficiently accelerating communication, they cannot take on the essential role of the neuroradiologists. Urgent and unexpected communication to pediatric patients and families cannot prescind from the human touch. Fear, discouragement, and loss of hope that patients may experience from the communication of a severe diagnosis must be addressed with human courage, professional assistance, and unwavering effort aiming at explaining the diagnosis, predicting the prognosis, and offering the best treatment to our little patients.

#### 2.1.6. Workflow Organization and Workload Distribution

A pediatric neuroradiology department is a kaleidoscopic microcosm characterized by procedures, rules, and delicate balances that allow an optimal workflow organization and a proper workload distribution to the final goal of offering the best healthcare to pediatric patients. Recently, most pediatric neuroradiology departments worldwide have suffered from neuroradiologists shortage and overwhelming workloads that risk impairing the quality of service and leading to workers’ burnout [29]. AI have demonstrated significant accuracy in supporting the department workflow organization and management and workload distribution, with a reduction in management costs [30]. Particularly, AI models are capable of creating dynamic shift plans based on department needs, patients’ availability, and neuroradiologists’ expertise. A tailored assignment of specific neuroradiological exams to neuroradiologists with specific experience in the field increases diagnostic accuracy and efficiency, but also promotes a workload distribution based on equity principles, fostering a pleasant workplace and actively fighting staff burnout. Moreover, an AI-powered, automated algorithm can intelligently modulate the number and type of shifts and workloads based on multiple variables, including staff pathologies, maternity leaves, and paid time off, ensuring continuous coverage and supporting work–life balance for pediatric neuroradiologists, which in turn benefits pediatric patients.

### 2.2. Current Clinical Applications in Pediatric Neuroradiology

#### 2.2.1. AI Implementation in Pediatric MRI Image Acquisition and Reconstruction, and Artefact Correction

The role of AI in imaging acquisition and image reconstruction is continuously expanding and aims to optimize imaging protocols to reduce radiation exposure, improve the quality of images, minimize the impact of artefacts, reduce the acquisition time, which is paramount considering the scarce pediatric tolerance to imaging, and cost-effective since it may support productivity and the imaging exam waiting lists.

##### MRI Image Acquisition and Reconstruction

**Figure 2:**
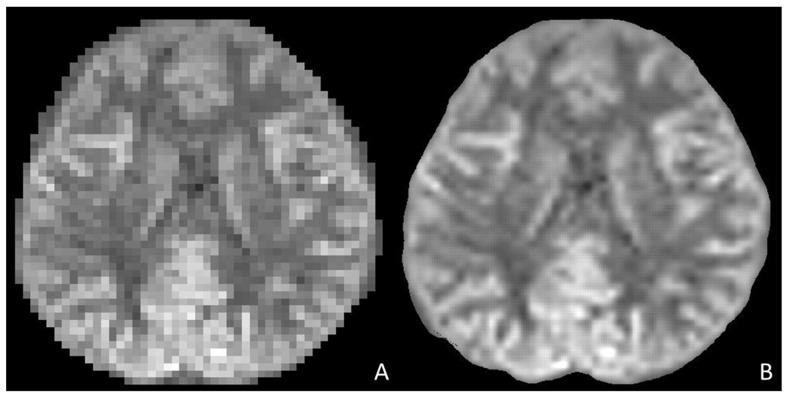
Compares an axial ASL-derived CBF map (**A**) and the same CBF map after the application of a super-resolution convolutional neural network (**B**) (required time: 0.5192 s), characterized by a significant improvement of image resolution (https://github.com/onnx/models (accessed on 20 July 2025)). The network was applied to the data, and the results were visualized using MATLAB (MATLAB ver. 24.1–R2024a [The MathWorks Inc. (2024a). MATLAB version: 24.1 (R2024a), Natick, MA, USA: The MathWorks Inc. https://www.mathworks.com (accessed on 20 July 2025)]). *ASL (Arterial Spin Labelling)*; *CBF (Cerebral Blood Flow)*.

**Figure 3:**
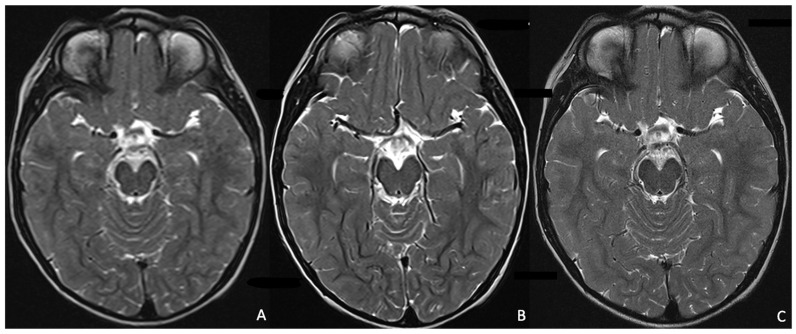
Shows the resolution improvement of a low-resolution brain T2WI acquisition (**A**) using Deep Resolve (**B**), an AI-powered image reconstruction technology based on convolutional neural networks [45], as compared to a high-resolution brain T2WI. The low-resolution T2WI (166 × 208 pixels) acquisition time is 58 s and was retrospectively enhanced by the Deep Resolve algorithm, which offers a high-resolution image (333 × 416 pixels) without extending the acquisition time and optimizing clinical workflow. In contrast, the high-resolution brain T2WI acquisition (350 × 350 pixels) shows similar resolution quality (**C**), but a longer acquisition time (5 min and 56 s). *WI (Weighted Imaging)*.

MRI acquisitions and the following reconstruction are extremely time-consuming and may delay crucial diagnosis with dire consequences to pediatric patients. Recent DL models, and particularly convolutional neural networks (CNNs), have proved to be promising in image reconstruction since CNNs can learn complex mappings between undersampled K-space data and high-quality images, enabling faster acquisition times. Particularly, zero-filling and super-resolution methods may improve image resolution and quality from undersampled data, reducing the acquisition time [31,32,33,34,35] (Figure 2). Indeed, obtaining high-resolution images by adjusting scanner protocol parameters during acquisition can result in prolonged scan times, which are often not optimal in clinical practice. In contrast, the use of deep learning–based post-processing methods allows for the enhancement of standard-resolution images, significantly reducing the required acquisition time (Figure 3) [36]. Also, combining compressed sensing and DL has led to accelerated MRI acquisition since DL algorithms can learn optimal regularization terms, improving image quality and reducing artefacts compared to traditional compressed sensing methods [37,38,39,40,41,42]. These models are unified by the common intent of reducing the acquisition time, yet offering high-quality, diagnostic MRI images. These models have been applied to most MRI sequences, including DWI/ADC (Diffusion Weighted-Image/Apparent Diffusion Coefficient), T1WI, and T2WI, demonstrating improved image quality and reduced scan times [43,44,45].

##### Motion Artefact Correction

Motion artefacts are among the most common and challenging artefacts in the pediatric population, especially in neonates. AI-based motion correction techniques reduce the need for children’s sedation thanks to automatic and robust algorithms. Particularly, DL algorithms enable the detection and quantification of patients’ motion, enabling the selection of optimal sequences [46,47,48,49] and the proper planning for motion correction, which can be retrospective or prospective. The retrospective motion correction protocols are commonly based on DL algorithms that improve the quality of already-acquired images [50,51,52]. On the other hand, the prospective motion correction protocols enable the prediction and compensation of motion before and during the acquisition, by reducing the need for repeating scans and ensuring optimal images [46,53,54]. Prospective and retrospective motion correction protocols can be combined to offer an exceptional MRI image quality result [52]. Although the applications of AI-based motion correction artefact algorithms encompass all the fields of radiology, this AI is paramount in pediatric neuroradiology since subtle morphological and functional change detection may be severely impaired by motion artefacts [55,56].

#### 2.2.2. Disease Classification, Prognostication, and Treatment Response Prediction

AI is a critical decision support tool for pediatric neuroradiologists, offering valuable assistance in classifying lesions, predicting prognosis and treatment response, and scheduling optimal patients’ follow-ups [12,27,57]. AI-based algorithms have demonstrated comparable to superior accuracy in basic imaging analysis to clinical experts. Moreover, AI algorithms have been used to classify pathologies and flag more complex cases for subspecialty consultation [58]. For example, Forestieri et al. applied machine learning and deep learning algorithms to analyze whole-body MRI acquisitions, successfully distinguishing chronic nonbacterial osteomyelitis lesions from normal bone marrow growth-related changes [59]. By integrating demographic, laboratory, imaging, and genetics data, AI models may predict patients’ prognosis and treatment response and offer insights into overall survival, progression-free survival, and neurodevelopmental outcomes [60,61]. In this regard, radiogenomics offers a thrilling correlation between imaging and genetics for different types of pediatric brain tumours to the pediatric neuroradiologist. These data support the differential diagnosis, suggest prognostication, and predict treatment response, contributing to the landscape of personalized medical approaches [1]. Treatment response is key in pediatric neuroradiology since it may help shape patients’ management and the optimal therapy timing and type. In addition, AI systems have been trained to automate patient follow-ups for significant incidental findings, ensuring that necessary steps are taken to address these issues, thereby improving patient outcomes and reducing potential liability [4]. The applications in neuroradiology are always expanding; therefore, we will focus on the most common ones.

##### Anatomical Segmentation and Quantitative Assessment in Pediatric Neuroradiology

**Figure 4:**
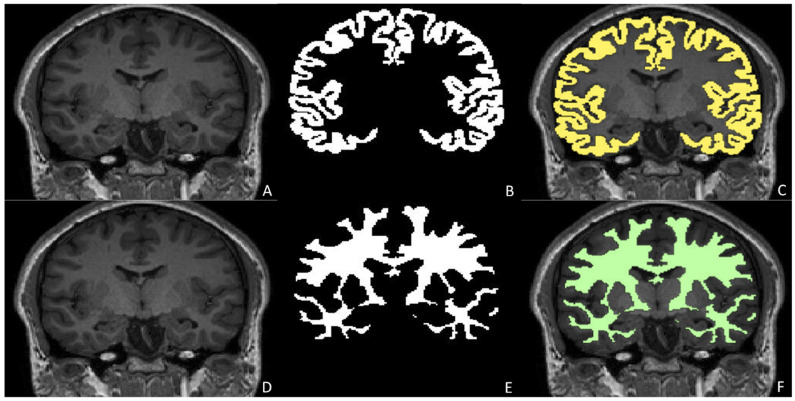
Shows the segmentation of brain tissues by SynthSeg, a pre-trained U-Net-based convolutional neural network on a coronal 3D T1 MPRAGE sequence (**A**,**D**) (required time: 10.35 s) [73]. Particularly, the segmented grey matter (**B**) and white matter (**E**) may be superimposed on the 3D T1 MPRAGE image (grey matter in yellow in (**C**), white matter in green in (**F**)). The network was applied to the data, and the results were visualized using MATLAB (MATLAB ver. 24.1–R2024a [The MathWorks Inc. (2024a). MATLAB version: 24.1 (R2024a), Natick, MA, USA: The MathWorks Inc. https://www.mathworks.com (accessed on 20 July 2025)]). *MPRAGE (Magnetisation Prepared Rapid Gradient Echo Imaging)*.

Accurate segmentation and quantitative assessment of anatomical and pathological structures are essential to enhance precision and consistency in evaluating brain structures and, therefore, to ensure optimal comparative analyses and disease serial characterization over time. These advancements are particularly significant in monitoring conditions such as hydrocephalus and assessing the impact of treatments on brain development. Manual segmentation is a long and bias-prone operation, and pediatric brain MRI presents unique challenges for segmentation due to the developmental variability of anatomical structures and the presence of motion artefacts [62,63] (Figure 4). Therefore, automated AI-supported segmentation represents the future in this field. DL-based segmentation through CNNs, particularly U-Net and V-net architectures, has state-of-the-art performance in medical image segmentation [64,65,66,67]. For instance, segmentation may be applied to anatomical structures, such as specific brain regions, and pathological lesions, such as brain tumours, with the possibility of comparing lesion volumes and therefore progression or regression after therapy and planning surgery [68,69,70,71]. Moreover, Grimm et al. demonstrated the effectiveness of a CNN in segmenting cerebrospinal fluid and brain volumes in pediatric patients affected by hydrocephalus, achieving a Dice coefficient of 0.86, indicating high accuracy in segmentation tasks [72].

##### Tumour Detection, Characterization, and Evolution

The 2021 World Health Organisation (WHO) classification of brain tumours completely divided adult and pediatric tumours since pediatric brain cancer exhibits a completely peculiar and wide range of molecular and genetic types with different pathogenesis and neuroradiological presentation at MRI [74,75]. This distinction underscores the need for specialized diagnostic approaches in pediatric neuro-oncology [76]. AI, specifically DL, has emerged as a pivotal tool in the detection, characterization, and monitoring of pediatric brain tumours, offering advancements in diagnostic accuracy, treatment planning, and prognostication. DL models have been demonstrated to be promising in the differential diagnosis of pediatric brain tumours, such as ependymoma, pilocytic astrocytoma, and medulloblastoma [77,78,79]. For instance, Das et al. used textural analysis to classify childhood medulloblastoma into WHO-defined subtypes, achieving high accuracy rates (>90%) [80]. Similarly, Li et al. developed a DL algorithm to differentiate pediatric intracranial germ cell tumour subtypes and predicted survival outcomes based on MRI data, named iGNet, which achieved high diagnostic performance, with area under the curve (AUC) values between 0.869 and 0.950 [81]. Voicu et al. adopted a machine learning algorithm in combination with Diffusion Kurtosis Imaging (DKI) for the discrimination of pediatric fossa tumour types in order to improve diagnostic accuracy and inform clinical decision-making [82]. Di Giannatale et al. adopted AI-based radiomics to non-invasively characterize neuroblastoma tumours through the prediction of the CT-obtained MYCN amplification status, a marker linked to prognosis and tumour behaviour [83]. These studies highlight the potential of AI in enhancing the precision of tumour classification, which is crucial for determining appropriate therapeutic strategies. Moreover, brain tumour AI-based automated segmentation has been proven to be time-saving as compared to manual segmentation, and accurate for volumetric analysis and treatment monitoring, providing consistent, and reproducible results for assessing tumour burden, planning surgical interventions, and monitoring treatment response [64,69,84,85]. Kazerooni et al. conducted a multi-institutional study demonstrating the effectiveness of DL models in automated tumour segmentation and brain tissue extraction from multiparametric MRI scans of pediatric brain tumours [85]. The differentiations among different subtypes of tumours and the precise definition of evolution and regression carry significant value due to the clinical and treatment implications on the pediatric patient’s life quality and overall survival [86].

##### Traumatic Brain Injury

Pediatric traumatic brain injury (TBI) is often an emergency and, overall, a severe condition that requires early diagnosis and guided clinical or surgical management. Proper and repeated neuroimaging is key to identifying primary injuries, such as fractures, hemorrhage and contusions, and secondary injuries, encompassing brain edema and herniation, which can evolve over time [87]. While some injuries, like hemorrhages or contusions, are easily detectable through standard CT and MRI, subtle and severe pathologies, like diffuse axonal injury (DAI), are challenging entities that require advanced MRI sequences and may be overlooked in an emergency context in which the time is limited, resources can be not immediately available, and the workload is constantly increasing worldwide [87]. ML and DL algorithms can automatically identify abnormalities, quantify lesion volumes, and detect patterns associated with TBI, with similar to higher accuracy as compared to pediatric neuroradiologists [88,89]. AI-based prognostication is based on the combined analysis of clinical and laboratory data, and multimodal neuroimaging, to predict long-term neurodevelopmental outcomes and suggest proper treatment and rehabilitation planning [88,90,91,92,93].

##### Congenital Brain Malformations

Congenital brain malformations (CBMs) encompass multiple structural abnormalities secondary to disruptions in normal brain development during pregnancy, ranging from neuronal migration disorders to midline anomalies like the Chiari and Dandy–Walker malformations, which can result in significant neurological deficits, developmental delays, and life-threatening complications [94]. The kaleidoscopic complexity of the CBM differential diagnosis is based on the huge variability in neuroradiological presentation, evolving imaging phenotypes over time, and overlaps between different malformation subtypes. Standard and advanced MRIs are interpreted by high-skilled pediatric neuroradiologists, who are frequently lacking in non-specialized institutions, and inter-observer variability is high [95,96]. DL algorithms have been applied to fetal and neonatal US and MRI exams, showing optimal accuracy, consistency, and efficiency to automatically detect and classify congenital CBMs [97,98]. Particularly, CNNs have been trained to differentiate between normal and abnormal brain structures, to identify specific malformations such as Chiari II malformation and Dandy–Walker complex, and to automatically measure brain structure to longitudinally monitor disease progression or post-surgical outcomes [99,100,101,102]. Finally, AI-based CBM prognostication can be crucial in prenatal counselling, postnatal care planning, and treatment strategies [103].

##### Epilepsy Detection and Pre-Surgical Planning

**Figure 5:**
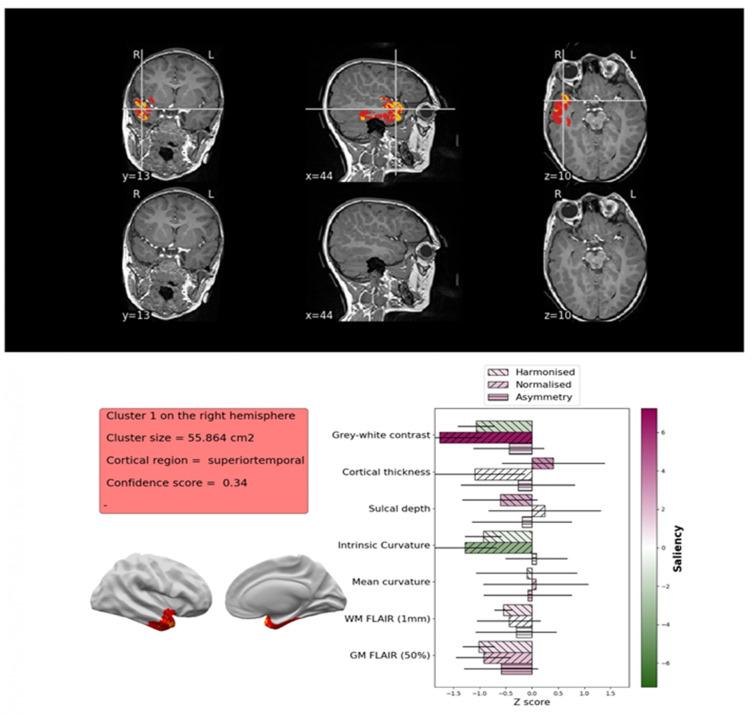
Patient’s MRI exam was processed through the MELD surface-based FCD detection algorithm, and a large FCD cluster was detected in the right temporal lobe. Particularly, the 3D T1 MPRAGE and FLAIR sequences were processed using FreeSurfer [120] to extract the following features: grey-white matter intensity contrast, cortical thickness, sulcal depth, intrinsic curvature, mean curvature, and FLAIR intensity sampled at different intracortical and subcortical depths. To ensure robustness, these features underwent several pre-processing steps. Specifically, harmonization was performed to correct for site- and scanner-related differences; normalization was performed to account for intra- and inter-subject variability; and asymmetry analysis was performed to enhance detection of inter-hemispheric differences. Prior to application on the patient, the MELD Graph U-Net model was trained on a large multi-centre dataset and benchmarked against an existing algorithm [121]. to enable precise identification of the lesion location, size, characteristics, and feature saliency, namely the relative importance of the MRI features. (https://github.com/MELDProject (accessed on 20 July 2025)) *MELD (Multi-centre Epilepsy Lesion Detection); FCD (Focal Cortical Dysplasia); MPRAGE (Magnetisation Prepared Rapid Gradient Echo Imaging), FLAIR (fluid-attenuated inversion recovery)*.

Pediatric epilepsy affects 0.5–1% of children worldwide, with significant impacts on their neurological development and quality of life. Therefore, identifying the underlying cause is essential for specific medical or surgical treatments [104]. Unfortunately, the identification of epileptogenic foci, which is usually performed with MRI, may be challenging since some foci may be difficult to identify, such as focal cortical dysplasia (FCD) or hippocampal sclerosis [105]. CNNs and U-Net architectures have been trained on large, annotated datasets to automatically localize epileptogenic lesions in pediatric populations [106,107,108,109]. Particularly, Ganji et al. trained an ML algorithm to identify FCD type IIb, which is a common cause of drug-resistant epilepsy in children [110]. The automated ML algorithm showed optimal sensitivity (96.7%), specificity (100%), and accuracy (98.6%) in FCD type IIb identification and diagnosis, demonstrating its efficiency in presurgical assessment and in improving postsurgical outcomes [110]. The Multicenter Epilepsy Lesion Detection (MELD) project (https://github.com/MELDProject (accessed on 20 July 2025)) developed a robust and interpretable deep-learning algorithm for the detection of FCD on a large multicentre MRI cohort of patients [111,112,113]. Through the MELD algorithm, it is possible to enhance the sensitivity of detecting subtle cortical abnormalities, which are often missed by conventional imaging but are relevant in clinical workflows (Figure 5). Moreover, pre-surgical planning is an emerging application of AI, which is used to provide quantitative lesion maps and spatial localisation of epileptogenic zones, which can be co-registered with electroencephalography (EEG) and magnetoencephalography (MEG) findings [114]. For instance, DL tools have been used to detect hippocampal sclerosis through automated volumetric and texture analyses [115,116] and to predict seizure and surgical outcomes, with optimal results [109,117,118,119].

##### White Matter Disorders

Pediatric white matter disorders encompass a huge pathology spectrum, including complex conditions like pediatric-onset multiple sclerosis (POMS) and metabolic leukodystrophies. AI-powered models allow for qualitative pattern recognition and quantitative biomarker discovery. For instance, an AI application for MRI analysis for subtle changes and pattern recognition is highly relevant to the early detection and monitoring of white matter lesions characteristic of MS (multiple sclerosis) [2]. ML algorithms applied to MRI tractography facilitated automated white matter connectivity analysis and were able to identify and characterize subtle abnormalities in fibre tracts [122], while DL models allowed automatic detection of white matter injuries and punctate white matter lesions in preterm infants [123,124]. Particularly, Zhu et al. presented an ultrasound data-driven diagnostic system for white matter injury in preterm infants, which combined multi-task DL and traditional radiomics features to achieve automatic detection of white matter regions, and to design a fusion strategy of DL features and manual radiomics features to obtain stable and efficient diagnostic performance [123]. Ultrasound radiomics diagnostic system achieved an AUC of 0.845 in the testing set. Meanwhile, the multi-task deep learning model showed a Dice coefficient of 0.78 in WM segmentation and AN AUC of 0.863 in the prediction of white matter injury risk characterized in the testing cohort [123]. An interactome-driven prioritization algorithm applied to whole-exome and whole-genome sequencing data has achieved a high diagnostic yield, even identifying novel disease-causing genes and phenotypes for heterogeneous genetic white matter disorders (GWMDs) [125]. DL models have been applied to prognostication, and particularly to forecast disease progression and severity in children affected by MS and metabolic leukodystrophies [2]. Finally, AI supports personalized medicine by integrating patients’ clinical, laboratory, and imaging data to suggest optimal patient management and tailored therapies [2].

##### Neurodevelopmental Disorders

Neurodevelopmental disorders in pediatric neuroradiology include autism spectrum disorder (ASD), attention-deficit/hyperactivity disorder (ADHD), and developmental delay, which are often associated with subtle and complex alterations in brain structure and connectivity [126,127,128]. ML and DL algorithms analyze structural MRI data to search for associated morphological and functional abnormalities, such as atypical cortical thickness, altered white matter integrity, or disrupted functional connectivity in children with ASD or ADHD [129]. Furthermore, AI-based approaches facilitate the integration of neuroimaging with genetic, behavioural, and clinical data, providing a more comprehensive understanding of the underlying biology of neurodevelopmental disorders and supporting personalized treatment planning. Finally, AI can also be used to evaluate the progression of these disorders and to assess the effectiveness of interventions [130,131,132,133,134].

## 3. Current Challenges and Ethical Considerations

The integration of AI into pediatric neuroradiology shows both huge potential and technical and ethical challenges that must be carefully addressed. These considerations span from data privacy, AI model transparency and generalizability, regulatory and implementation hurdles, as well as legal responsibility.

### 3.1. Data Privacy and Security

The use of pediatric patients’ medical data requires high standards of privacy and confidentiality. AI models, especially those using large-scale neuroimaging datasets, strongly depend on the collection, storage, and sharing of sensitive patients’ information, with the constant risks of data breaches and unauthorized accesses [135]. Robust data anonymization, differential privacy techniques, and independent oversight are essential for protecting pediatric patients’ data [136].

### 3.2. Informed Consent and Pediatric Autonomy

Ethical challenges also include how to ensure proper and informed consent for pediatric patients’ data use in AI-based research. Commonly, parents express their consent for diagnostic and therapeutic procedures of pediatric patients. On the other hand, older children may want to assent or dissent regarding the use of their imaging data. Finally, the use of old MRI scans for AI model training should be taken into consideration for a secondary use without explicit consent, which should be ethically justified by institutional review boards [137].

### 3.3. Algorithmic Transparency and Explainability

AI models should be intelligible to pediatric neuroradiologists to let them understand and supervise how data are analyzed and results are obtained. The “black box” nature of some DL models raises accountability and trustworthiness concerns [138]. Recent studies advocate for the development of intelligible AI systems by the use of techniques like saliency maps, feature attribution, and model-agnostic methods to provide pediatric neuroradiologists with clear explanations [139]. It is paramount that AI models offer accurate, consistent and repeatable results, obtained through explainable paths and always under the supervision of pediatric neuroradiologists, who can actively shape the models through feedback or changes to the data or algorithms. Apart from the specific techniques and systems, diagnostic, prognostic and surgical decisions in pediatric neuroradiology need to be supported by AI, but preserve the human touch.

### 3.4. Bias and Representativeness, Robustness and Generalizability of AI Algorithms

Data scarcity and inhomogeneity, and the absence of large, well-annotated datasets are major hurdles in AI implementation in pediatric neuroradiology. Pediatric neuroimaging data are limited due to disease rarity, ethical concerns, and challenges associated with acquiring high-quality exams in pediatric patients. The lack of standardized imaging protocols and the absence of specific and univocal annotation guidelines lead to dataset heterogeneity with a deep impact on AI models’ performances and a lack of results’ generalizability. These issues hamper the creation of AI algorithms that are tailored for pediatric patients [140]. Moreover, pediatric patients range from neonates to teenagers, who exhibit a wide range of developmental stages, leading to significant variability in brain anatomy and pathology presentation. ML and DL models trained on datasets that underrepresent certain populations, based on age, ethnicity, disease type, or imaging modality, demonstrate reduced performance when applied to data acquired in different clinical environments. Possible solutions may be represented by transfer learning, image-to-image translation and AI-based augmentation of datasets. In pediatric neuroradiology, where diseases may manifest differently than in adults, transfer learning can lead to inaccurate or missed diagnoses due to the different MRI characteristics of pediatric and adult populations [141,142]. Image-to-image translation has shown good results since AI proved to be able to generate synthetic CT images from MRI data [20], synthetic PET images from contrast-enhanced MRI [24]. AI augmentation of datasets thanks to advanced generative adversarial networks has proved to be promising in tackling the small pediatric datasets issues [143]. On the other hand, synthetic imaging still faces regulatory hurdles, and concerns among clinicians, which should be promptly addressed. In conclusion, to ensure fairness, equity and inclusivity, datasets must be diverse, well-labelled, and representative of the full pediatric population.

### 3.5. Regulatory and PACS Implementation Hurdles

The regulatory landscape for AI in healthcare is constantly evolving, and obtaining approval for AI tools requires compliance with rigorous standards to ensure patient safety and efficacy [144]. Moreover, implementing AI solutions necessitates perfect integration with Picture Archiving and Communication Systems (PACS) and Electronic Health Records (EHRs), which represents a technical challenge and a need for workflow adjustments [144]. In this regard, AI vendors are called to develop user-friendly interfaces which easily communicate with PACS. Finally, the financial implications of developing, implementing, and maintaining AI systems are enormous. Healthcare institutions should invest in AI tools, considering key parameters such as improved diagnostic accuracy, workflow efficiency, and patient outcomes [144].

### 3.6. Clinical Responsibility and Human Oversight

AI should be viewed as an augmented intelligence tool, enhancing the pediatric neuroradiologist’s decision-making rather than replacing it. AI’s role must remain assistive, with ultimate responsibility resting with pediatric neuroradiologists, who should be involved in the design, validation, and deployment of AI models to ensure clinical relevance and safety [145,146].

## 4. Future Perspectives

### 4.1. Federated Learning

Federated learning (FL) is a pivotal opportunity to address pediatric patients’ data scarcity while supporting collaborative AI model training. FL allows AI models to learn from various data from multiple institutions without compromising patients’ privacy and enhances AI model robustness and generalizability in pediatric neuroradiology. FL demonstrated its efficiency in multi-institutional brain imaging analyses, underscoring its potential in pediatric settings. Particularly, Raggio et al. introduced FedSynthCT-Brain, which employs a cross-silo horizontal FL approach that allows multiple centres to collaboratively train a U-Net-based deep learning model to obtain synthetic brain CT images from MRI images [147]. The integration of multi-modal data, such as neuroimaging, clinical records, and molecular and genetic information, offers a wide perspective on pediatric neurological conditions. Such integration enhances diagnostic accuracy and supports patient-tailored treatment planning, as demonstrated by recent literature employing multi-modal approaches in neurodevelopmental disorder assessments [148]. The real-world applications of FL in pediatric neuroradiology are still limited, but may represent address the need for pediatric-specific big data and AI models.

### 4.2. Collaborative Research and AI Development

Collaboration among pediatric neuroradiologists, neuroscientists, and physicians is paramount for the successful and efficient implementation of AI into pediatric neuroradiology. AI models applied to clinical routine should be user-friendly, intelligible and aim at patients’ benefits [149]. standardized protocols and evaluation metrics are crucial for assessing AI model performance, refining AI models’ deficiencies, increasing algorithms’ generalizability, and facilitating regulatory approval. Creating representative datasets and validation frameworks is necessary to promote consistency and reliability in AI applications [150]. In conclusion, the future of AI in pediatric neuroradiology is promising and ongoing research, technological innovation, and collaborative efforts are key to efficiently implementing AI in pediatric neuroradiology.

## 5. Conclusions

Pediatric neuroradiology’s new era is enhanced by AI, which has been shown to be able to augment human expertise, redefine diagnostic precision, and potentially transform the way pediatric neurological disorders are diagnosed, prognosticated, and treated. AI’s effective integration in pediatric neuroradiology’s everyday routine will require strategic investments in research, education, and infrastructure, as well as a strong commitment to ethical, equitable, and patient-centred implementation. The application of artificial intelligence in pediatric neuroradiology does not replace the human touch, but amplifies human intellect and extends our capacity to diagnose, prognosticate, and treat with unprecedented precision and speed.

## Data Availability

Not applicable.

## Abbreviations

AI (artificial intelligence), DL (deep learning), ML (machine learning), CNN (convolutional neural network), WI (weighted image), DWI (diffusion weighted image), ADC (apparent diffusion coefficient), MRI (magnetic resonance imaging), CT (computed tomography), US (ultrasound), FL (federated learning), WHO (World Health Organisation), AUC (area under curve), TBI (pediatric traumatic brain injury), DAI (diffuse axonal injury), CBM (congenital brain malformation), FCD (focal cortical dysplasia), MELD (Multicenter Epilepsy Lesion Detection) EEG (electroencephalography), MEG (magnetoencephalography), ASD (autism spectrum disorder), ADHD (attention-deficit/hyperactivity disorder), POMS (pediatric-onset multiple sclerosis), MS (multiple sclerosis), GWMDs (genetic white matter disorders), PACS (Picture Archiving and Communication Systems), EHRs (Electronic Health Records), FLAIR (fluid-attenuated inversion recovery), MPRAGE (Magnetization Prepared Rapid Gradient Echo Imaging).
